# Improved conditions for the generation of beta oscillations in the subthalamic nucleus-globus pallidus network

**DOI:** 10.1186/1471-2202-13-S1-P116

**Published:** 2012-07-16

**Authors:** Alex Pavlides, S John Hogan, Rafal Bogacz

**Affiliations:** 1 Bristol Centre for Complexity Sciences and Department of Computer Science, University of Bristol, Bristol, UK; 2 Department of Engineering Mathematics, University of Bristol, Bristol, UK; 3 Department of Computer Science, University of Bristol, Bristol, UK

## 

A key pathology in the development of Parkinson’s disease is the occurrence of persistent beta oscillations, which are correlated with the difficulty of movement initiation. We investigate the network model composed of subthalamic nucleus (STN) and globus pallidus (GP) developed by Nevado Holgado *et al*. [1] who identified the conditions under which this circuit could generate beta oscillations. Our work extends their analysis by deriving improved analytic stability conditions for realistic values of the synaptic transmission delay between STN and GP neurons. For the range of synaptic transmission delays measured experimentally, the improved conditions are significantly closer to the results of simulations. Furthermore, our analysis explains how changes in cortical and striatal input to the STN-GP network influence oscillations generated by the circuit. Since we have identified when a system of mutually connected populations of excitatory and inhibitory neurons can generate oscillations, our results may also find applications in the study of neural oscillations produced by assemblies of excitatory and inhibitory neurons in other brain regions.
